# Are perceived bad working conditions and perceived workplace bullying associated with doctor visits? Results of the nationally representative German General Social Survey

**DOI:** 10.1186/s12913-019-4570-7

**Published:** 2019-10-15

**Authors:** André Hajek, Hans-Helmut König

**Affiliations:** Department of Health Economics and Health Services Research, Hamburg Center for Health Economics, University Medical Center Hamburg-Eppendorf, Martinistraße 52, 20246 Hamburg, Germany

**Keywords:** Health care utilization, Andersen’s behavioral model, Workplace, Bullying, Mobbing

## Abstract

**Background:**

The reason for doctor visits associated with bad working conditions (and workplace bullying) remains unknown. Therefore, the aim of this study was to examine the association between perceived working conditions as well as workplace bullying and the number of doctor visits as well as the reason for seeing a doctor.

**Methods:**

Data were derived from the German General Social Survey, a representative cross-section of the population in the year 2014. Self-reported doctor visits in the last 3 months were used as outcome measure. Self-rated working conditions (noise, bad air; time/performance pressure; bad working atmosphere; overtime; shifts/night work; hard physical labour) and workplace bullying were assessed. The reason for seeing a doctor was also recorded (acute illness; chronic illness; feeling unwell; requesting advice; visit to the doctor’s office without consulting the doctor (e.g., need to get a prescription); preventive medical check-up/vaccination). Regression analysis stratified by sex was conducted.

**Results:**

Adjusting for various potential confounders, Poisson regressions showed that workplace bullying was associated with increased doctor visits in men, but not in women. Contrarily, time/performance pressure at work was only associated with increased doctor visits in women, but not in men. Furthermore, the probability of visiting the doctor for reasons of acute illness or feeling unwell increased with workplace bullying in men. The probability of visiting the doctor because of feeling unwell increased with time/performance pressure in women.

**Conclusions:**

Our findings stress the association between adverse working conditions (workplace bullying as well as time/performance pressure at work) and doctor visits, with remarkable gender differences. Longitudinal studies are required to confirm the present findings and to obtain further insights into this relationship.

## Background

### Literature and aims of this study

The determinants of outpatient physician visits were examined by various studies [1]. As theoretical framework, Andersen’s behavioral model was used by many researchers, distinguishing between predisposing characteristics (e.g., age or sex), enabling resources (e.g., access to health care) or need factors (e.g., chronic illnesses). It has, for example, been shown that chronic conditions are positively associated with doctor visits [2].

However, thus far, only a few studies investigated the association between *working conditions* as well as *workplace bullying* and doctor visits. Working conditions refer to “the working environment and aspects of an employee’s terms and conditions of employment” [3]. More specifically, most of the existing studies focused on the association between occupational stress and health care use [4–8], predominantly showing that increased job stress is associated with higher doctor visits. For example, Manning et al. showed that stressful work events and strain are positively associated with health care use among individuals from two different industries (*n* = 260) [5]. Using a nationally representative sample of the Canadian population (*n* = 29,110), another study [4] also showed that medium or high job strain was positively associated with the number of general practitioner (GP) or specialist visits. Negative associations between beneficial job characteristics (e.g., autonomy) and doctor visits over the past 3 months were found (*n* = 232 state civil service employees of the University of South Florida). Or, to put it the other way around, a higher degree of autonomy was associated with fewer doctor visits. Using staff from Monash University (Australia, *n* = 1925), a further study found that persons with lower job stress had fewer visits to a medical practitioner [7]. However, Raggatt [6] showed that work stress events were not associated with doctor visits in the past 12 months among male long-distance coach drivers in Australia (*n* = 93).

With regard to workplace bullying, − the second key independent variable of this study - some studies exist showing that it is associated with subsequent *sickness absence* [9]. However, to the best of our knowledge, studies are missing explicitly focusing on the relation between workplace bullying and *doctor visits*.

In sum, most of the respective studies used very *specific samples* that cannot be generalized to the general population. Moreover, previous studies mainly focused on the association between job stress and doctor visits *in general*. Thus, the first aim of the present study was to

1. investigate the association between perceived working conditions as well as perceived workplace bullying and doctor visits using a nationally representative sample in Germany.

Moreover, thus far, the *reason* for the doctor visits associated with bad working conditions remains unknown. Thus, a second aim of the current study was

(2) to examine the association between working conditions as well as workplace bullying and the reason for seeing a doctor (e.g., acute illness, chronic illness or sleep disorders).

### Rationale of the study

This knowledge may be beneficial to gain further insights into the link between bad working conditions and doctor visits. In addition, it has been shown that sex matters in health care use [1]. Therefore, regression analyses stratified by sex were conducted.

Knowing the association between working conditions/workplace bullying and doctor visits in general might help to underline the economic burden of adverse working conditions and workplace bullying for the health care system. This might be of importance because it has been shown that stress and coping workplace programs can help to reduce illness and health care use [10]. Moreover, knowing the factors associated with the reason for seeing a doctor might shed light into the nature of this relationship.

Knowledge regarding the link between workplace bullying and doctor visits might be of particular importance because workplace bullying is a frequent phenomenon [11] which also restricts workplace productivity [12].

### Possible mechanisms

At first glance, it appears plausible that workplace bullying is associated with more doctor visits as bullying at the workplace is, for example, associated with the risk for decreased self-rated health [13] or mental health complaints [14]. It has also been shown that bullied respondents have a higher body-mass-index (BMI) and are less physically active than non-bullied respondents [11]. Moreover, bullying is positively associated with being a current smoker [11]. However, evidence also exists revealing that individuals suffering from workplace bullying increase their efforts at work, for example to show their commitment and avoid being stigmatized once again [15]. As suggested by Nabe-Nielsen et al. [16], this might explain why workplace bullying is positively associated with sickness presentism (working while ill) [17]. Thus, one could assume that workplace bullying is associated with fewer doctor visits. Nevertheless, we assume that these presentism effects did not compensate for the substantial adverse health consequences of workplace bullying. Consequently, and in line with the studies examining the association between workplace bullying and sickness absence [9], we hypothesize that workplace bullying is associated with more doctor visits.

With regard to bad working conditions, it appears plausible that bad working conditions are associated with more doctor visits. This association might be explained by various factors. First, doctor visits might be used as a way to cope with stress or bad working conditions [4]. Moreover, work-related stress is associated with various health-risk behaviors, resulting in increased health care use. For example, it has been shown that work-related stress is associated with a higher BMI [18]. This can result in metabolic syndrome for reasons of low physical activity and increased energy intake [18]. It has also been demonstrated that higher levels of perceived stress are associated with more fast-food consumption and a greater intake of high-fat snacks [19]. Furthermore, it has been reported that high levels of perceived stress were associated with a low level of physical activity [20]. Moreover, and maybe the most important link, bad working conditions are associated with physical and mental illnesses [4]. For example, it has been demonstrated that it is associated with coronary heart disease [21] or headaches [22]. In turn, physical and mental illnesses are linked to an increased health care use [1].

### German health care system

Concerning the health care use, it is of importance to know key features of the health care system in Germany. It should be emphasized that health insurance is compulsory in Germany. While approximately 10% of the German population are insured by private health insurances (PHI), the remaining 90% of the German population are insured by social statutory health insurance (SHI) funds. In Germany, employees above an income-threshold, civil servants and self-employed persons can choose between PHI and SHI. Most types of outpatient treatment are covered by PHI as well as SHI. All insured individuals have access to comprehensive health care services. In Germany, the waiting time for outpatient physician appointments is typically short [23]. With regard to the health care system in Germany, more details are provided elsewhere [24].

## Methods

### Sample

For the present study, data were derived from the cross-sectional German General Social Survey (ALLBUS). The ALLBUS-Surveys have been conducted biennially since 1980. The data consist of independent random samples drawn from community-dwelling respondents aged 18 and over. The ALLBUS samples were extended to the former East Germany (new Federal States) and to foreigners who speak German (with a residence in Germany). Generally, about 3000 to 3500 individuals take part in the ALLBUS survey. It is one of the most often used data sets in social sciences in Germany. A broad range of topics is covered in the ALLBUS surveys. For example, questions on attitudes (e.g., attitudes towards welfare state) or opinions (e.g., opinions on immigrants) are included. Moreover, demographic data were gathered. Computer-assisted personal interviews were conducted. Further details are, for example, provided by Terwey [25].

In the year 2014 (from March to September; *n* = 3471), special emphasis was placed on health variables (e.g., doctor visits). Consequently, we used this wave [26]. The response rate was 35.0% in this wave. The main reason for non-participation was refusal. The response rate is similar to other large survey studies conducted in Germany [27] and reflects the trend of declining participation rate in Germany [28]. Please see the strengths and limitations section for further details.

The age range in our analytical sample is similar to that of the working age in Germany (unrestricted from 18 years onwards). The current retirement age in Germany is about 65. However, the retirement age is to be increased continuously and reach 67 years (by 2029). However, since some individuals living in Germany also work after retirement, we decided to include these individuals in our analytical sample. This means that individuals were covered from 18 to 74 years. Nevertheless, it may be worth noting that about 99% of the individuals included in regression analysis were aged 18 to 64 years.

An ethical statement for the ALLBUS surveys was not required since the criteria for such a statement were not fulfilled (for instance, examination of patients, risk for the respondents, lack of information about the goals of the study, use of invasive methods). This is in accordance with national regulations. Prior to the interview, written informed consent was given by all participants of the study.

### Outcome variables

Self-reported doctor visits in the last 3 months were used as outcome measure. In addition, the reason for seeing a doctor in the past 3 months was also recorded for individuals reporting at least one doctor visit in the last 3 months (no or yes; multiple responses possible):

- acute illness (e.g, flu, injury)
- chronic illness (e.g., diabetes, high blood pressure/hypertension, rheumatism)
- felt unwell (e.g., general discomfort, sleep disorders)
- requesting advice
- visit to the doctor’s office, but without consulting the doctor (e.g., need to get a prescription, radiotherapy)
- preventive medical check-up/vaccination

### Independent variables

#### Working conditions

Working conditions (“Now we would like to learn more about the working conditions at your main job. Is your current job/occupational activity strongly, somewhat or not at all characterised by A Noise, dust, gases, vapours, bad air; B Time/performance pressure; C A bad working atmosphere; D Overtime, long working hours; E Shifts or night work; F Hard physical labour”) and workplace bullying were assessed. The working conditions were assessed on a 3-point scale (yes, strongly; yes, somewhat; no, not at all). Workplace bullying was assessed as follows: “How often do you feel unfairly criticised, bullied or embarrassed in front of others by colleagues or superiors?” (often; sometimes; seldom; never).

#### Covariates

In the current study, covariates were selected based on Andersen’s behavioral model. Predisposing factors were used as follows: age, marital status (0 = married and living apart; widowed; divorced; never married; civil partnership, living apart; registered partner deceased; civil partnership dissolved; 1 = married and living together with spouse; civil partnership, living together) and educational level (ISCED-97 [29]), distinguishing between basic education, lower secondary, upper secondary, post secondary, higher tertiary, and upper tertiary. With regard to enabling resources (e.g., (perceived) access to health care), covariates were not included in this study due to reasons of data availability.

Regarding the need factors, self-rated impairments in activities of daily living and chronic conditions were used. Impairments were rated as follows: “When you climb stairs, i.e. go up several floors on foot: Does your state of health affect you greatly, slightly or not at all”? and “And what about having to cope with other tiring everyday tasks, e.g. lifting something heavy or performing tasks requiring agility: Does your state of health affect you greatly, slightly or not at all?” (both: greatly; slightly; not at all). Chronic conditions were assessed using the total number of chronic conditions (allergy; migraine; high blood pressure, hypertension; circulatory disorder of the heart, angina pectoris; rheumatism, chronic inflammation of the joints, arthritis, arthrosis, gout; spinal damage; chronic bronchitis; asthma; inflammation of the liver, hepatitis, liver shrinkage, liver cirrhosis; diabetes; cancer; osteoporosis). With regard to the lifestyle factors, the current smoking status (no; yes), and BMI categories (self-rated and according to the World Health Organization thresholds: underweight: BMI < 18.5 kg/m^2^, normal weight: 18.5 kg/m^2^ ≤ BMI < 25 kg/m^2^, overweight: 25 kg/m^2^ ≤ BMI < 30 kg/m^2^, and obesity: BMI ≥ 30 kg/m^2^) were used.

### Statistical analysis

First, descriptive sample statistics and bivariate associations were computed. Second, the determinants of the number of doctor visits in the preceding 3 months were examined using multiple Poisson regression models with cluster-robust standard errors. This analytical choice is in accordance with other recent studies investigating the determinants of the number of doctor visits [30–32]. Third, the determinants of the other six outcome measures (reason for doctor visits: acute illness; chronic illness; felt unwell; requesting advice; visit to the doctor’s office, but without consulting the doctor; preventive medical check-up/vaccination) were analyzed using multiple logistic regressions. As there is evidence that gender matters in health care use [1], regression analysis was performed stratified by sex. Most of the missing values can simply be explained by the fact that working conditions were only assessed when the individuals are currently part- or full-time employed.

The level of significance was set at α = .05. Statistical analysis was performed using Stata Release 14.2 (Stata Corp., College Station, Texas).

## Results

### Sample characteristics

Table 1 shows the sample characteristics for the individuals included in Poisson regressions stratified by sex. In both sexes, average age was about 43 years (ranging from 18 to 74 years). In both sexes, about 40% of the individuals had strong time/performance pressure in his or her job and approximately 15% of the individuals had experienced workplace bullying ‘sometimes’ or ‘often’. While the average number of doctor visits was 1.4 (±2.3) in men, it equaled 2.1 (±3.7) in women. Among the individuals visiting a doctor in the preceding 3 months, acute illnesses was the most common reason. Further details are displayed in Table 1.

**Table 1:** Sample characteristics for the individuals included in Poisson regressions stratified by sex

	Men (*n* = 1041)	Women (*n* = 818)
Age: Mean (SD); Range	43.5 (12.4); 18–74	43.2 (11.5); 18–73
Married, living together with spouse/partner: N (%)	614 (59.0%)	446 (54.5%)
Education (ISCED-97): N (%)		
° Basic education	7 (0.7%)	4 (0.5%)
° Lower secondary	62 (5.9%)	45 (5.5%)
° Upper secondary	468 (45.0%)	336 (41.1%)
° Post secondary	61 (5.9%)	99 (12.1%)
° Higher tertiary	419 (40.2%)	317 (38.7%)
° Upper tertiary	24 (2.3%)	17 (2.1%)
Weight category: N (%)		
° Underweight	4 (0.4%)	25 (3.0%)
° Normal weight	401 (38.5%)	445 (54.4%)
° Overweight	447 (42.9%)	237 (29.0%)
° Obese	189 (18.2%)	111 (13.6%)
Currently smoking: N (%)	390 (37.5%)	224 (27.4%)
Number of chronic diseases: Mean (SD); Range	0.8 (1.0); 0–6	1.0 (1.2); 0–8
Working conditions		
Noise, dust, gases, vapours, bad air: N (%)		
° yes, strongly	246 (23.6%)	107 (13.1%)
° yes, somewhat	293 (28.2%)	175 (21.4%)
° no, not at all	502 (48.2%)	536 (65.5%)
Time/performance pressure: N (%)		
° yes, strongly	423 (40.6%)	316 (38.6%)
° yes, somewhat	473 (45.5%)	369 (45.1%)
° no, not at all	145 (13.9%)	133 (16.3%)
Bad working atmosphere: N (%)		
° yes, strongly	45 (4.3%)	32 (3.9%)
° yes, somewhat	262 (25.2%)	206 (25.2%)
° no, not at all	734 (70.5%)	580 (70.9%)
Overtime, long working hours: N (%)		
° yes, strongly	246 (23.6%)	125 (15.3%)
° yes, somewhat	447 (43.0%)	373 (45.6%)
° no, not at all	348 (33.4%)	320 (39.1%)
Shifts or night work: N (%)		
° yes, strongly	129 (12.4%)	86 (10.5%)
° yes, somewhat	177 (17.0%)	67 (8.2%)
° no, not at all	735 (70.6%)	665 (81.3%)
Hard physical labour: N (%)		
° yes, strongly	159 (15.3%)	80 (9.8%)
° yes, somewhat	256 (25.6%)	168 (20.5%)
° no, not at all	626 (60.1%)	570 (69.7%)
Workplace bullying: N (%)		
° often	28 (2.7%)	34 (4.2%)
° sometimes	119 (11.4%)	97 (11.8%)
° seldom	299 (28.7%)	219 (26.8%)
° never	595 (57.2%)	468 (57.2%)
Impairments: Climb stairs: N (%)		
° Greatly affected	32 (3.1%)	36 (4.4%)
° Slightly affected	178 (17.1%)	179 (21.9%)
° Not at all affected	831 (79.8%)	603 (73.7%)
Impairments: Everyday tasks: N (%)		
° Greatly affected	56 (5.4%)	56 (6.8%)
° Slightly affected	247 (23.7%)	210 (25.7%)
° Not at all affected	738 (70.9%)	552 (67.5%)
Number of doctor visits: Mean (SD); Range; Proportion of individuals with no doctor visits	1.4 (2.3); 0–30; 38.1%	2.1 (3.7); 0–65; 29.2%
Reason for doctor visit (yes): Acute illness: N (%)	242 (37.6%)	209 (36.2%)
Reason for doctor visit (yes): Chronic illness: N (%)	120 (18.6%)	117 (20.2%)
Reason for doctor visit (yes): Felt unwell: N (%)	49 (7.6%)	57 (9.9%)
Reason for doctor visit (yes): Requesting advice: N (%)	72 (11.2%)	53 (9.2%)
Reason for doctor visit (yes): visit to the doctor’s office, but without consulting the doctor: N (%)	79 (12.3%)	89 (15.4%)
Reason for doctor visit (yes): preventive medical check-up/vaccination: N (%)	167 (25.9%)	209 (36.2%)

The reason for doctor visit sum up to 644 (men) and 578 (women)

### Bivariate associations: working conditions and (reason for) doctor visits

In *men*, doctor visits in general were only associated with workplace bullying (r = −.15, bonferroni-adjusted significance level *p* < .001). Moreover, the probability of a doctor visit for reason of acute illness was associated with ‘noise, dust, gases, vapours, bad air’ (χ^2^(2) = 6.05, *p* = .049) and shifts or night work (χ^2^(2) = 7.00, *p* = .030). The probability of a doctor visit because of feelings of malaise was associated with workplace bullying (χ^2^(3) = 9.10, *p* = .028). Furthermore, the probability of a doctor visit (visit to the doctor’ office, but without consulting the doctor) was associated with time/performance pressure (χ^2^(2) = 6.20, *p* = .045). The probability of a doctor visit for reason of requesting advice was associated with overtime, long working hours (χ^2^(2) = 7.28, *p* = .026). The probability of a doctor visit for reason of preventive medical check-up/vaccination was associated with shifts or night work (χ^2^(2) = 6.30, *p* = .043).

In *women*, doctor visits in general were only associated with time/performance pressure (r = −.09, bonferroni-adjusted significance level *p* = .039). In addition, the probability of a doctor visit for reason of chronic illness was associated with shifts or night work (χ^2^(2) = 8.29, *p* = .016) and hard physical labour (χ^2^(2) = 12.15, *p* = .002). The probability of a doctor visit because of feelings of malaise was associated with time/performance pressure (χ^2^(2) = 10.49, *p* = .005) and workplace bullying (χ^2^(3) = 12.13, *p* = .007). Furthermore, the probability of a doctor visit (visit to the doctor’ office, but without consulting the doctor) was associated with a bad working atmosphere (χ^2^(2) = 9.33, *p* = .009). The probability of a doctor visit for reason of preventive medical check-up/vaccination was associated with hard physical labour (χ^2^(2) = 7.54, *p* = .023).

### Regression analysis

Table 2 shows the determinants of doctor visits in the preceding 3 months in men (first column) and women (second column). For reasons of clarity, the control variables are not displayed in Table 2 and Table 3. Regression tables where “no, not at all” (working conditions) and “never” (workplace bullying) were used as reference categories are displayed in Additional file 1: Table S1 and Additional file 2: Table S2, respectively.

**Table 2:** Determinants of doctor visits in the preceding 3 months. Results of Poisson regressions (first column: men; second column: women)

	(1)	(2)
Independent variables	Doctor visits - Men	Doctor visits - Women
Control variables	✓	✓
Noise, dust, gases, vapours, bad air: - yes, somewhat (Ref.: yes, strongly)	0.04	−0.08
	(0.14)	(0.15)
- no, not at all	0.07	− 0.08
	(0.16)	(0.15)
Time/performance pressure: - yes, somewhat (Ref.: yes, strongly)	−0.16	− 0.20+
	(0.10)	(0.11)
- no, not at all	0.03	−0.50**
	(0.15)	(0.17)
Bad working atmosphere: - yes, somewhat (Ref.: yes, strongly)	0.36	−0.06
	(0.26)	(0.27)
- no, not at all	0.38	−0.20
	(0.29)	(0.29)
Overtime, long working hours: - yes, somewhat (Ref.: yes, strongly)	0.00	−0.16
	(0.11)	(0.19)
- no, not at all	0.07	−0.04
	(0.12)	(0.20)
Shifts or night work: - yes, somewhat (Ref.: yes, strongly)	−0.08	− 0.14
	(0.18)	(0.22)
- no, not at all	−0.07	0.04
	(0.16)	(0.16)
Hard physical labour: - yes, somewhat (Ref.: yes, strongly)	−0.08	0.31+
	(0.15)	(0.18)
- no, not at all	−0.00	0.47*
	(0.17)	(0.20)
Workplace bullying: - Sometimes (Ref.: often)	−0.64*	0.36
	(0.30)	(0.25)
- Seldom	−1.12***	0.29
	(0.29)	(0.25)
- Never	−1.04***	0.28
	(0.29)	(0.25)
Constant	0.83*	1.75***
	(0.41)	(0.47)
Observations	1041	818
Pseudo R^2^	0.141	0.178

All estimates include age, marital status, educational level, smoking status, BMI category, impairments (climb stairs; everyday tasks) and morbidity as potential confounders. Poisson coefficients were reported; cluster-robust standard errors in parentheses; *** *p* < 0.001, ** *p* < 0.01, * *p* < 0.05, + *p* < 0.10

**Table 3:** Determinants of visiting the doctor (for various reasons). Results of logistic regressions (1 = yes, visiting the doctor for this reason; 0 = otherwise)

Independent variables	(1)	(2)	(3)	(4)	(5)	(6)	(7)	(8)	(9)	(10)	(11)	(12)
	Reason for doctor visit: Acute illness - Men	Reason for doctor visit: Acute illness - Women	Reason for doctor visit: Chronic illness – Men	Reason for doctor visit: Chronic illness - Women	Reason for doctor visit: Felt unwell - Men	Reason for doctor visit: Felt unwell - Women	Reason for doctor visit: Requesting advice - Men	Reason for doctor visit: Requesting advice - Women	Reason for doctor visit: Visit to the doctor’s office (without consulting the doctor) - Men	Reason for doctor visit: Visit to the doctor’s surgery (without consulting the doctor) - Women	Reason for doctor visit: Preventive medical check-up/vaccination – Men	Reason for doctor visit: Preventive medical check-up/vaccination - Women
Control variables	✓	✓	✓	✓	✓	✓	✓	✓	✓	✓	✓	✓
Noise, dust, gases, vapours, bad air: - yes, somewhat (Ref.: yes, strongly)	0.54*	1.20	0.68	0.73	0.37*	1.02	1.55	1.01	1.10	1.89	1.55	1.04
	(0.32–0.90)	(0.64–2.25)	(0.35–1.32)	(0.33–1.62)	(0.16–0.89)	(0.34–2.99)	(0.64–3.75)	(0.37–2.76)	(0.52–2.31)	(0.79–4.55)	(0.87–2.75)	(0.53–2.01)
- no, not at all	0.56*	0.91	0.97	0.53+	0.62	1.15	1.87	1.09	1.49	1.72	0.95	1.33
	(0.33–0.96)	(0.51–1.63)	(0.48–1.97)	(0.25–1.10)	(0.27–1.45)	(0.42–3.10)	(0.71–4.96)	(0.42–2.81)	(0.71–3.11)	(0.74–3.99)	(0.52–1.75)	(0.73–2.40)
Time/performance pressure: - yes, somewhat (Ref.: yes, strongly)	1.10	0.87	0.69	1.36	0.91	0.37**	1.64	0.55+	1.42	0.55*	0.66+	1.04
	(0.73–1.65)	(0.56–1.35)	(0.40–1.19)	(0.73–2.52)	(0.39–2.12)	(0.18–0.75)	(0.89–3.02)	(0.27–1.11)	(0.76–2.64)	(0.32–0.94)	(0.42–1.02)	(0.68–1.61)
- no, not at all	1.89*	0.98	0.65	0.88	1.67	0.32*	0.87	0.93	0.75	0.83	0.61	0.64
	(1.07–3.34)	(0.53–1.81)	(0.31–1.35)	(0.37–2.12)	(0.56–4.99)	(0.11–0.95)	(0.32–2.41)	(0.34–2.48)	(0.31–1.84)	(0.38–1.84)	(0.32–1.17)	(0.33–1.22)
Bad working atmosphere: - yes, somewhat (Ref.: yes, strongly)	0.94	0.64	1.22	1.67	0.78	1.26	0.88	2.80	0.98	7.81*	1.78	2.21
	(0.39–2.25)	(0.26–1.57)	(0.41–3.62)	(0.40–6.97)	(0.24–2.60)	(0.32–4.96)	(0.27–2.83)	(0.30–25.83)	(0.21–4.51)	(1.06–57.77)	(0.52–6.14)	(0.75–6.54)
- no, not at all	0.75	0.61	1.26	3.02	0.82	1.24	0.60	2.65	1.89	3.61	2.65	2.48+
	(0.31–1.83)	(0.25–1.49)	(0.42–3.84)	(0.67–13.62)	(0.23–2.93)	(0.29–5.26)	(0.19–1.93)	(0.29–24.00)	(0.40–8.82)	(0.51–25.47)	(0.75–9.44)	(0.84–7.30)
Overtime, long working hours: - yes, somewhat (Ref.: yes, strongly)	1.30	1.29	0.85	0.54	0.82	1.42	0.35**	0.70	1.78	1.19	1.04	0.97
	(0.82–2.06)	(0.73–2.29)	(0.44–1.62)	(0.25–1.20)	(0.34–2.01)	(0.53–3.79)	(0.17–0.72)	(0.32–1.54)	(0.82–3.87)	(0.55–2.56)	(0.64–1.72)	(0.55–1.69)
- no, not at all	1.15	1.32	1.16	0.77	1.08	1.41	0.34**	0.52	1.48	1.63	0.87	1.00
	(0.69–1.94)	(0.71–2.45)	(0.56–2.40)	(0.33–1.79)	(0.41–2.85)	(0.49–4.05)	(0.16–0.73)	(0.21–1.30)	(0.63–3.46)	(0.76–3.49)	(0.49–1.54)	(0.56–1.81)
Shifts or night work: - yes, somewhat (Ref.: yes, strongly)	0.99	1.15	0.89	0.80	2.02	0.87	0.71	0.61	0.72	0.97	0.83	0.53
	(0.51–1.94)	(0.44–3.05)	(0.39–2.01)	(0.22–2.87)	(0.73–5.62)	(0.16–4.83)	(0.27–1.88)	(0.08–4.40)	(0.28–1.84)	(0.31–3.07)	(0.37–1.85)	(0.21–1.34)
- no, not at all	0.62	1.50	1.04	0.48	0.86	1.28	0.67	1.54	0.81	1.14	1.64	0.49+
	(0.35–1.10)	(0.73–3.07)	(0.51–2.13)	(0.19–1.25)	(0.33–2.19)	(0.46–3.57)	(0.30–1.51)	(0.45–5.22)	(0.35–1.86)	(0.47–2.73)	(0.81–3.31)	(0.23–1.04)
Hard physical labour: - yes, somewhat (Ref.: yes, strongly)	0.85	1.59	1.13	1.11	1.92	0.71	0.75	1.03	2.02	0.98	1.35	1.16
	(0.47–1.53)	(0.74–3.44)	(0.50–2.55)	(0.37–3.29)	(0.70–5.25)	(0.24–2.06)	(0.31–1.77)	(0.28–3.84)	(0.80–5.11)	(0.37–2.65)	(0.69–2.64)	(0.52–2.59)
- no, not at all	1.03	1.52	1.58	1.62	2.14	1.33	0.78	1.30	2.40*	0.57	0.97	1.56
	(0.55–1.95)	(0.71–3.25)	(0.66–3.75)	(0.57–4.58)	(0.78–5.87)	(0.51–3.46)	(0.30–2.01)	(0.37–4.55)	(1.01–5.70)	(0.19–1.65)	(0.48–1.98)	(0.69–3.53)
Workplace bullying: - Sometimes (Ref.: often)	0.32*	0.91	0.70	1.37	0.39	2.13	3.04	1.96	0.96	0.58	0.98	0.68
	(0.11–0.91)	(0.35–2.34)	(0.20–2.42)	(0.39–4.77)	(0.10–1.52)	(0.45–10.00)	(0.52–17.88)	(0.20–18.79)	(0.18–5.01)	(0.13–2.51)	(0.26–3.70)	(0.23–2.02)
- Seldom	0.33*	0.86	0.47	0.88	0.25*	0.97	2.00	1.26	0.47	1.69	1.24	1.04
	(0.12–0.91)	(0.34–2.14)	(0.14–1.57)	(0.24–3.21)	(0.07–0.97)	(0.19–4.86)	(0.35–11.54)	(0.12–13.32)	(0.09–2.38)	(0.45–6.30)	(0.34–4.54)	(0.37–2.89)
- Never	0.30*	0.72	0.59	0.98	0.26+	0.64	2.77	2.20	0.53	1.42	1.08	0.83
	(0.11–0.82)	(0.28–1.81)	(0.17–2.02)	(0.25–3.88)	(0.07–1.02)	(0.13–3.25)	(0.48–15.83)	(0.23–21.58)	(0.11–2.60)	(0.36–5.68)	(0.29–3.99)	(0.29–2.37)
Constant	18.07**	3.69	0.10*	0.03**	0.40	0.06+	0.02*	0.04	0.00***	0.02**	0.03***	0.14+
	(2.90–112.56)	(0.56–24.55)	(0.01–0.74)	(0.00–0.37)	(0.03–5.23)	(0.00–1.10)	(0.00–0.37)	(0.00–1.97)	(0.00–0.04)	(0.00–0.36)	(0.00–0.21)	(0.02–1.24)
Observations	645	572	642	577	645	577	642	561	642	577	645	579
Pseudo R^2^	0.083	0.068	0.160	0.253	0.109	0.104	0.093	0.064	0.131	0.087	0.059	0.052

All estimates include age, marital status, educational level, smoking status, BMI category, impairments (climb stairs; everyday tasks) and morbidity as potential confounders. Odds Ratios were reported; 95%-confidence intervals in parentheses; *** *p* < 0.001, ** *p* < 0.01, * *p* < 0.05, + *p* < 0.10

Adjusting for several covariates, Poisson regressions revealed that workplace bullying was associated with increased doctor visits in men (Ref.: often; sometimes: β = −.64, *p* = .031; seldom: β = − 1.12, *p* < .001; never: β = − 1.04, p < .001), but not in women (with significant gender differences; not shown here). Contrarily, time/performance pressure was only associated with increased doctor visits in women (Ref.: yes, strongly; yes, somewhat: β = −.20, *p* = .059; no, not at all: β = −.50, *p* = .004), but not in men (with significant gender differences; not shown here).

As for the control variables (not shown here, but available upon request), impairments (climb stairs and everyday tasks) were associated with increased doctor visits in women, whereas the number of chronic diseases was associated with increased doctor visits in men. Further details are available upon request.

For individuals reporting at least one doctor visit in the preceding 3 months, the reason for seeing a doctor in the last 3 months was recorded (no or yes; multiple responses possible; please see the methods section for further details). Thus, the determinants of six different outcome variables are depicted in Table 3 (reason for doctor visit): (1) acute illness (e.g, flu, injury), (2) chronic illness (e.g., diabetes, high blood pressure/hypertension, rheumatism), (3) feeling unwell (e.g., general discomfort, sleep disorders), (4) requesting advice, (5) visit to the doctor’s office, but without consulting the doctor (e.g., need to get a prescription, radiotherapy), (6) preventive medical check-up/vaccination. Please note that the OR and the 95%-CI are not displayed in the text for reasons of clarity. Please see Table 3 for further details.

Among other things, the probability of visiting the doctor for reasons of acute illness or because of feeling unwell increased with workplace bullying as well as ‘noise, dust, gases, vapours, bad air’ in men. However, gender differences were only significant for workplace bullying (results not shown, but available upon request). The probability of visiting the doctor because of feeling unwell increased with time/performance pressure in women (with significant gender differences; results not shown, but available upon request). Please see Table 3 for further details. As for the control variables, for example, the number of chronic diseases was associated with doctor visits for reasons of acute or chronic illnesses (not shown here). Further details are available upon request.

## Discussion

### Main findings

The current study aimed at identifying the association between working conditions and doctor visits in general using a nationally representative sample. A second aim of the current study was to examine the association between working conditions as well as workplace bullying and the reason for seeing a doctor. Adjusting for various covariates, Poisson regressions showed that workplace bullying was associated with increased doctor visits in men, but not in women (with significant gender differences). Contrarily, time/performance pressure at work was only associated with increased doctor visits in women, but not in men (with significant gender differences). Furthermore, the probability of visiting the doctor for reasons of acute illness or because of feeling unwell increased with workplace bullying in men. The probability of visiting the doctor because of feelings of malaise increased with time/performance pressure in women.

### Possible explanations

We found that workplace bullying was associated with more doctor visits in men. This extends knowledge from a recent systematic review [9] which concluded that workplace bullying is a risk factor for later sickness absence. For example, based on 9520 female employees in the Danish elder-care services, a previous study [33] has shown that workplace bullying is associated with long-term sickness absence after adjusting for various potential confounders such as psychosocial work conditions.

In addition, the probability of visiting the doctor for reasons of acute illness or because of feeling unwell increased with workplace bullying in men in our study. Consequently, we assume that workplace bullying is associated with feelings of malaise in men, but not in women. This association, in general, appears plausible because a recent study [11] has shown that workplace bullying is associated with lack of restful sleep and awaking problems. Possible reasons for these gender differences might be that men experiencing workplace bullying might fear negative consequences on their career prospects or they might fear subsequent unemployment. It has, for example, been shown that unemployment has long-lasting negative effects on subjective well-being in men, but not in women in Germany [34]. This has also been shown for other countries [35, 36].

In our study, time/performance pressure at work was only associated with increased doctor visits in women, but not in men. Moreover, we found that the probability of visiting the doctor because of feelings of malaise increased with time/performance pressure in women. This appears plausible because time and performance pressure in women can interfere with obligations in other areas (such as family obligations, commitments to friends/relatives or volunteer and community work). This has also been reported in previous studies [37–39]. Thus, pressure might be a barrier to healthy eating and physical activity [40] which in turn are associated with factors such as self-esteem or sleep quality [41]. We think that our findings are in line with a recent longitudinal study based on data from the German Socio-Economic Panel (GSOEP), which shows that increasing working hours also increases the number of doctor visits in women [42]. They also assume that the dual home (paid work and in home) may explain this link.

However, future studies are required to clarify why workplace bullying is linked to feelings of malaise when visiting the doctor only in men. Moreover, future research is needed to clarify why time/performance pressure is related to feelings of malaise only in women.

### Strengths and limitations

Data for our study were derived from a large, nationally representative sample (Germans and foreigners) in 2014. Self-reported doctor visits in the last 3 months were used as outcome measure, mitigating recall bias [43]. In addition, the reasons for doctor visits were explored, markedly reducing the “black box” of doctor visits. In the present study, main adverse working conditions (e.g., time/performance pressure; bad working atmosphere, shifts/night work; hard physical labour) and workplace bullying were covered. A single item measure was used to quantify workplace bullying. Therefore, our findings should be confirmed using other validated instruments [44]. Moreover, the association between other adverse working conditions (e.g., high risk of accidents at work; stereotypical activities) and doctor visits should be explored. Furthermore, subgroup analyses (e.g., for physicians or nursing staff) should be conducted in future studies. Because of the relatively low response rate, the potential for sample selection bias exists. Therefore, we cannot dismiss the possibility that the observed associations will underestimate the true association. However, intense efforts were done to reduce this potential problem in the German General Social Survey [45]. In addition, the present study is a cross-sectional one. Such a study has widely acknowledged limitations (e.g., regarding the causality). For example, it is not possible to study changes within individuals over time. Furthermore, it should be acknowledged that individuals judged their working conditions (self-reported data). Thus, we cannot dismiss the possibility that self-rated working conditions did not fully reflect actual working conditions. Therefore, the external validity may be limited. This should be addressed in future studies.

## Conclusion

The findings of the current study emphasize the association between adverse working conditions (workplace bullying as well as time/performance pressure at work) and doctor visits, with remarkable gender differences. Longitudinal studies are needed to confirm the present findings and to obtain further insights into the temporal relationship between these variables.

## Supplementary information

**Additional file 1: Table S1.** Determinants of doctor visits in the preceding three months. Results of Poisson regressions (first column: men; second column: women).

**Additional file 2: Table S2.** Determinants of visiting the doctor (for various reasons). Results of logistic regressions (1 = yes, visiting the doctor for this reason; 0 = otherwise).

## Data Availability

The data used in this study are third-party data. The anonymized data sets of the ALLBUS are available for secondary analysis for researchers. ALLBUS documentation materials and data sets are downloadable for free from the GESIS Data Catalog (DBK). To download data sets, individuals are required to create a GESIS-account. Please see for further details: https://www.gesis.org/en/en/allbus/download/

## Abbreviations

ALLBUS: German General Social Survey
BMI: Body-mass-index
CI: Confidence interval
GP: General practitioner
ISCED: International Standard Classification of Education
OR: Odds ratio
PHI: Private health insurance
SHI: Statutory health insurance

## References

[1] Babitsch B, Gohl D, von Lengerke T (2012). Re-revisiting Andersen’s Behavioral Model of Health Services Use: a systematic review of studies from 1998–2011. GMS Psychosoc Med.

[2] Hajek A, Bock J, König H (2017). Which factors affect health care use among older Germans? Results of the German ageing survey. BMC Health Serv Res.

[3] Eurofound. Working conditions [https://www.eurofound.europa.eu/topic/working-conditions]. Accessed 9 Oct 2019.

[4] Azagba S, Sharaf MF (2011). Psychosocial working conditions and the utilization of health care services. BMC Public Health.

[5] Manning MR, Jackson CN, Fusilier MR (1996). Occupational stress and health care use. J Occup Health Psychol.

[6] Raggatt PTF (1991). Work stress among long-distance coach drivers: a survey and correlational study. J Organ Behav.

[7] Sharpley CF, Reynolds R, Acosta A, Dua JK (1996). The presence, nature and effects of job stress on physical and psychological health at a large Australian university. J Educ Adm.

[8] Spector PE, Jex SM (1991). Relations of job characteristics from multiple data sources with employee affect, absence, turnover intentions, and health. J Appl Psychol.

[9] Nielsen MB, Indregard A-MR, Øverland S (2016). Workplace bullying and sickness absence: a systematic review and meta-analysis of the research literature. Scand J Work Environ Health.

[10] Rahe RH, Taylor CB, Tolles Robbyn L, Newhall Lynn M, Veach Tracy L, Susan B (2002). A novel stress and coping workplace program reduces illness and healthcare utilization. Psychosom Med.

[11] Hansen ÅM, Gullander M, Hogh A, Persson R, Kolstad HA, Willert MV, Bonde JP, Kaerlev L, Rugulies R, Grynderup MB (2016). Workplace bullying, sleep problems and leisure-time physical activity: a prospective cohort study. Scand J Work Environ Health.

[12] Fattori A, Neri L, Aguglia E, Bellomo A, Bisogno A, Camerino D, Carpiniello B, Cassin A, Costa G, De Fazio P (2015). Estimating the impact of workplace bullying: humanistic and economic burden among workers with chronic medical conditions. Biomed Res Int.

[13] Bonde JP, Gullander M, Hansen ÅM, Grynderup M, Persson R, Hogh A, Willert MV, Kaerlev L, Rugulies R, Kolstad HA (2016). Health correlates of workplace bullying: a 3-wave prospective follow-up study. Scand J Work Environ Health.

[14] Einarsen S, Nielsen MB (2015). Workplace bullying as an antecedent of mental health problems: a five-year prospective and representative study. Int Arch Occup Environ Health.

[15] Hoel H, Sheehan MJ, Cooper CL, Einarsen S, Einarsen S, Hoel H, Zapf D, Cooper C (2011). Organisational effects of workplace bullying. Bullying and harassment in the workplace: Developments in theory, research, and practice.

[16] Nabe-Nielsen K, Grynderup MB, Conway PM, Clausen T, Bonde JP, Garde AH, Hogh A, Kaerlev L, Török E, Hansen ÅM (2017). The role of psychological stress reactions in the longitudinal relation between workplace bullying and turnover. J Occup Environ Med.

[17] Conway PM, Clausen T, Hansen ÅM, Hogh A (2016). Workplace bullying and sickness presenteeism: cross-sectional and prospective associations in a 2-year follow-up study. Int Arch Occup Environ Health.

[18] Ippoliti F, Corbosiero P, Canitano N, Massoni F, Ricciardi M, Ricci L, Archer T, Ricci S (2017). Work-related stress, over-nutrition and cognitive disability. La Clinica terapeutica.

[19] Barrington WE, Beresford SA, McGregor BA, White E (2014). Perceived stress and eating behaviors by sex, obesity status, and stress vulnerability: findings from the vitamins and lifestyle (VITAL) study. J Acad Nutr Diet.

[20] Barrington WE, Ceballos RM, Bishop SK, McGregor BA, Beresford SA. Peer reviewed: perceived stress, behavior, and body mass index among adults participating in a worksite obesity prevention program, Seattle, 2005–2007. Prev Chronic Dis. 2012;9:E152. 10.5888/pcd9.120001 PMC3477899 23036611

[21] Twisk JW, Snel J, Kemper HC, van Mechelen W (1999). Changes in daily hassles and life events and the relationship with coronary heart disease risk factors: a 2-year longitudinal study in 27–29-year-old males and females. J Psychosom Res.

[22] De Benedittis G, Lorenzetti A (1992). The role of stressful life events in the persistence of primary headache: major events vs. daily hassles. Pain.

[23] Zok K (2007). Warten auf den Arzttermin. Ergebnisse einer Repräsentativumfrage unter GKV-und PKV-Versicherten. WIdO-monitor.

[24] Passon A, Lüngen M, Gerber A. et al. Das Krankenversicherungssystem in Deutschland. In: Lauterbach K, Stock S, Brunner H. Hrsg. Gesundheitsökonomie. Bern: Verlag Hans Huber. 2013;105-136.

[25] Terwey M (2000). ALLBUS: a German general social survey. Schmollers Jahr.

[26] GESIS - Leibniz-Institut für Sozialwissenschaften: Allgemeine Bevölkerungsumfrage der Sozialwissenschaften ALLBUS 2016. GESIS Datenarchiv, Köln. ZA5250 Datenfile Version 2.1.0. 2017. 10.4232/1.12796.

[27] Neller K (2005). Kooperation und Verweigerung. Eine non-response-Studie [co-operation and refusal: a non-response study]. ZUMA Nachrichten.

[28] Stoop I, Billiet J, Koch A, Fitzgerald R (2010). Improving survey response: lessons learned from the European social survey.

[29] UNESCO (2006). International Standard Classification of Education. ISCED 1997, Re-edition.

[30] Hajek A, Brettschneider C, Eisele M, Lühmann D, Mamone S, Wiese B, Weyerer S, Werle J, Fuchs A, Pentzek M, Stein J, Luck T, Weeg D, Mösch E, Heser K, Scherer M, Maier W, Riedel-Heller SG, König HH. Does transpersonal trust moderate the association between chronic conditions and general practitioner visits in the oldest old? Results of the AgeCoDe and AgeQualiDe study. Geriatr Gerontol Int. 2019;19(8):705–10. 10.1111/ggi.13693 31237101

[31] Ortega AN, McKenna RM, Pintor JK, Langellier BA, Roby DH, Pourat N, Bustamante AV, Wallace SP (2018). Health care access and physical and behavioral health among undocumented Latinos in California. Med Care.

[32] Pinilla J, Rodriguez-Caro A (2019). Differences in healthcare utilisation between users and non-users of homeopathic products in Spain: results from three waves of the National Health Survey (2011-2017). PLoS One.

[33] Clausen T, Hogh A, Borg V (2012). Acts of offensive behaviour and risk of long-term sickness absence in the Danish elder-care services: a prospective analysis of register-based outcomes. Int Arch Occup Environ Health.

[34] Clark AE, Diener E, Georgellis Y, Lucas RE (2008). Lags and leads in life satisfaction: A test of the baseline hypothesis. Econ J.

[35] Clark AE, Georgellis Y (2013). Back to baseline in Britain: adaptation in the British household panel survey. Economica.

[36] Rudolf R, Kang S-J (2015). Lags and leads in life satisfaction in Korea: when gender matters. Fem Econ.

[37] Goux D, Maurin E, Petrongolo B (2014). Worktime regulations and spousal labor supply. Am Econ Rev.

[38] Paull G (2008). Children and women's hours of work. Econ J.

[39] Sparks K, Cooper C, Fried Y, Shirom A (1997). The effects of hours of work on health: a meta-analytic review. J Occup Organ Psychol.

[40] Welch N, McNaughton SA, Hunter W, Hume C, Crawford D (2009). Is the perception of time pressure a barrier to healthy eating and physical activity among women?. Public Health Nutr.

[41] Fox KR (1999). The influence of physical activity on mental well-being. Public Health Nutr.

[42] Cygan-Rehm K, Wunder C (2018). Do working hours affect health? Evidence from statutory workweek regulations in Germany. Labour Econ.

[43] Bhandari A, Wagner T (2006). Self-reported utilization of health care services: improving measurement and accuracy. Med Care Res Rev.

[44] Cowie H, Naylor P, Rivers I, Smith PK, Pereira B (2002). Measuring workplace bullying. Aggr Violent Behav.

[45] Wasmer M, Blohm M, Walter J, Scholz E, Jutz R (2014). Konzeption und Durchführung der “Allgemeinen Bevölkerungsumfrage der Sozialwissenschaften” (ALLBUS) 2012.

