# Supplementary material for: Are perceived bad working conditions and perceived workplace bullying associated with doctor visits? Results of the nationally representative German General Social Survey
Source: BMC Health Serv Res. 2019 Oct 15;19:697. doi: 10.1186/s12913-019-4570-7 (PMC6794812; doi:10.1186/s12913-019-4570-7)
Supplement: Supplementary file 1 — Additional file 1: Table S1. Determinants of doctor visits in the preceding three months. Results of Poisson regressions (first column: men; second column: women). [file 12913_2019_4570_MOESM1_ESM.docx]

Additional file Table 1: Determinants of doctor visits in the preceding three months. Results of Poisson regressions (first column: men; second column: women).

|  | (1) | (2) |
| --- | --- | --- |
| Independent variables | Doctor visits - Men | Doctor visits - Women |
|  |  |  |
| Control variables | 🗸 | 🗸 |
|  |  |  |
| Noise, dust, gases, vapours, bad air: - yes, strongly (Ref.: no, not at all) | -0.07 | 0.08 |
|  | (0.16) | (0.15) |
| - yes, somewhat | -0.02 | -0.00 |
|  | (0.12) | (0.11) |
| Time/performance pressure: - yes, strongly (Ref.: no, not at all) | -0.03 | 0.50** |
|  | (0.15) | (0.17) |
| - yes, somewhat | -0.19 | 0.29* |
|  | (0.14) | (0.14) |
| Bad working atmosphere: - yes, strongly (Ref.: no, not at all) | -0.38 | 0.20 |
|  | (0.29) | (0.29) |
| - yes, somewhat | -0.02 | 0.14 |
|  | (0.11) | (0.13) |
| Overtime, long working hours: - yes, strongly (Ref.: no, not at all) | -0.07 | 0.04 |
|  | (0.12) | (0.20) |
| - yes, somewhat | -0.06 | -0.12 |
|  | (0.10) | (0.10) |
| Shifts or night work: - yes, strongly (Ref.: no, not at all) | 0.07 | -0.04 |
|  | (0.16) | (0.16) |
| - yes, somewhat | -0.01 | -0.17 |
|  | (0.12) | (0.19) |
| Hard physical labour: - yes, strongly (Ref.: no, not at all) | 0.00 | -0.47* |
|  | (0.17) | (0.20) |
| - yes, somewhat | -0.08 | -0.16 |
|  | (0.13) | (0.12) |
| Workplace bullying: - Often (Ref.: never) | 1.04*** | -0.28 |
|  | (0.29) | (0.25) |
| - Sometimes | 0.39* | 0.08 |
|  | (0.18) | (0.14) |
| - Seldom | -0.08 | 0.01 |
|  | (0.09) | (0.11) |
| Constant | 0.26 | 1.72*** |
|  | (0.33) | (0.47) |
|  |  |  |
| Observations | 1,041 | 818 |
| Pseudo R² | 0.141 | 0.178 |

All estimates include age, marital status, educational level, smoking status, BMI category, impairments (climb stairs; everyday tasks) and morbidity as potential confounders. Poisson coefficients were reported; cluster-robust standard errors in parentheses; *** p<0.001, ** p<0.01, * p<0.05, + p<0.10
