# Supplementary material for: Are perceived bad working conditions and perceived workplace bullying associated with doctor visits? Results of the nationally representative German General Social Survey
Source: BMC Health Serv Res. 2019 Oct 15;19:697. doi: 10.1186/s12913-019-4570-7 (PMC6794812; doi:10.1186/s12913-019-4570-7)
Supplement: Supplementary file 2 — Additional file 2: Table S2. Determinants of visiting the doctor (for various reasons). Results of logistic regressions (1 = yes, visiting the doctor for this reason; 0 = otherwise). [file 12913_2019_4570_MOESM2_ESM.docx]

Additional file Table 2: Determinants of visiting the doctor (for various reasons). Results of logistic regressions (1 = yes, visiting the doctor for this reason; 0 = otherwise).

|  | (1) | (2) | (3) | (4) | (5) | (6) | (7) | (8) | (9) | (10) | (11) | (12) |
| --- | --- | --- | --- | --- | --- | --- | --- | --- | --- | --- | --- | --- |
| Independent variables | Reason for doctor visit: Acute illness - Men | Reason for doctor visit: Acute illness - Women | Reason for doctor visit: Chronic illness – Men | Reason for doctor visit: Chronic illness - Women | Reason for doctor visit: Felt unwell - Men | Reason for doctor visit: Felt unwell - Women | Reason for doctor visit: Requesting advice - Men | Reason for doctor visit: Requesting advice - Women | Reason for doctor visit: Visit to the doctor’s office (without consulting the doctor) - Men | Reason for doctor visit: Visit to the doctor’s surgery (without consulting the doctor) - Women | Reason for doctor visit: Preventive medical check-up/vaccination – Men | Reason for doctor visit: Preventive medical check-up/vaccination - Women |
|  |  |  |  |  |  |  |  |  |  |  |  |  |
| Control variables | 🗸 | 🗸 | 🗸 | 🗸 | 🗸 | 🗸 | 🗸 | 🗸 | 🗸 | 🗸 | 🗸 | 🗸 |
|  |  |  |  |  |  |  |  |  |  |  |  |  |
| Noise, dust, gases, vapours, bad air: - yes, strongly (Ref.: no, not at all) | 1.78* | 1.10 | 1.03 | 1.89 | 1.60 | 0.87 | 0.53 | 0.92 | 0.67 | 0.58 | 1.05 | 0.75 |
|  | (1.02 - 3.11) | (0.62 - 1.95) | (0.51 - 2.06) | (0.88 - 4.06) | (0.64 - 4.04) | (0.35 - 2.18) | (0.22 - 1.32) | (0.35 - 2.42) | (0.30 - 1.52) | (0.25 - 1.36) | (0.57 - 1.92) | (0.42 - 1.36) |
| - yes, somewhat | 0.96 | 1.31 | 0.70 | 1.39 | 0.60 | 0.89 | 0.83 | 0.93 | 0.74 | 1.10 | 1.62* | 0.78 |
|  | (0.61 - 1.53) | (0.83 - 2.07) | (0.39 - 1.27) | (0.75 - 2.56) | (0.24 - 1.48) | (0.41 - 1.91) | (0.42 - 1.64) | (0.43 - 2.03) | (0.38 - 1.42) | (0.61 - 1.98) | (1.01 - 2.60) | (0.49 - 1.24) |
| Time/performance pressure: - yes, strongly (Ref.: no, not at all) | 0.53* | 1.02 | 1.54 | 1.14 | 0.60 | 3.13* | 1.14 | 1.08 | 1.33 | 1.20 | 1.63 | 1.56 |
|  | (0.29 - 0.96) | (0.55 - 1.90) | (0.71 - 3.32) | (0.48 - 2.69) | (0.22 - 1.65) | (1.03 - 9.51) | (0.40 - 3.28) | (0.41 - 2.87) | (0.53 - 3.30) | (0.53 - 2.71) | (0.84 - 3.18) | (0.82 - 2.97) |
| - yes, somewhat | 0.58+ | 0.89 | 1.05 | 1.55 | 0.55 | 1.17 | 1.87 | 0.59 | 1.88 | 0.66 | 1.07 | 1.63+ |
|  | (0.34 - 1.01) | (0.50 - 1.57) | (0.52 - 2.15) | (0.70 - 3.43) | (0.22 - 1.39) | (0.39 - 3.50) | (0.71 - 4.96) | (0.23 - 1.51) | (0.83 - 4.25) | (0.30 - 1.42) | (0.58 - 1.99) | (0.91 - 2.92) |
| Bad working atmosphere: - yes, strongly (Ref.: no, not at all) | 1.32 | 1.65 | 0.79 | 0.33 | 1.22 | 0.81 | 1.67 | 0.38 | 0.53 | 0.28 | 0.38 | 0.40 |
|  | (0.55 - 3.22) | (0.67 - 4.07) | (0.26 - 2.37) | (0.07 - 1.56) | (0.30 - 4.98) | (0.19 - 3.34) | (0.50 - 5.58) | (0.04 - 3.18) | (0.13 - 2.24) | (0.03 - 2.25) | (0.11 - 1.25) | (0.14 - 1.20) |
| - yes, somewhat | 1.24 | 1.05 | 0.96 | 0.55+ | 0.96 | 1.02 | 1.47 | 1.06 | 0.52+ | 2.17* | 0.67+ | 0.89 |
|  | (0.81 - 1.91) | (0.64 - 1.74) | (0.54 - 1.70) | (0.27 - 1.12) | (0.42 - 2.19) | (0.47 - 2.20) | (0.77 - 2.80) | (0.46 - 2.45) | (0.26 - 1.04) | (1.16 - 4.04) | (0.42 - 1.08) | (0.54 - 1.47) |
| Overtime, long working hours: - yes, strongly (Ref.: no, not at all) | 0.87 | 0.76 | 0.86 | 1.31 | 0.93 | 0.71 | 2.96** | 1.93 | 0.68 | 0.61 | 1.15 | 1.00 |
|  | (0.51 - 1.48) | (0.41 - 1.41) | (0.44 - 1.70) | (0.59 - 2.88) | (0.37 - 2.37) | (0.26 - 1.93) | (1.37 - 6.38) | (0.76 - 4.93) | (0.29 - 1.60) | (0.28 - 1.37) | (0.65 - 2.06) | (0.54 - 1.83) |
| - yes, somewhat | 1.13 | 0.98 | 0.73 | 0.71 | 0.77 | 1.00 | 1.03 | 1.36 | 1.20 | 0.73 | 1.20 | 0.96 |
|  | (0.74 - 1.71) | (0.64 - 1.48) | (0.42 - 1.26) | (0.40 - 1.25) | (0.36 - 1.63) | (0.51 - 1.99) | (0.53 - 2.01) | (0.66 - 2.81) | (0.66 - 2.18) | (0.42 - 1.27) | (0.76 - 1.91) | (0.64 - 1.45) |
| Shifts or night work: - yes, strongly (Ref.: no, not at all) | 1.60+ | 0.67 | 0.96 | 2.06 | 1.17 | 0.78 | 1.49 | 0.65 | 1.24 | 0.88 | 0.61 | 2.03+ |
|  | (0.92 - 2.80) | (0.33 - 1.36) | (0.44 - 2.08) | (0.83 - 5.11) | (0.39 - 3.46) | (0.25 - 2.47) | (0.65 - 3.40) | (0.19 - 2.26) | (0.51 - 3.03) | (0.36 - 2.14) | (0.31 - 1.18) | (0.99 - 4.19) |
| - yes, somewhat | 1.59+ | 0.77 | 0.85 | 1.64 | 2.36* | 0.68 | 1.06 | 0.40 | 0.90 | 0.85 | 0.51* | 1.08 |
|  | (0.98 - 2.57) | (0.37 - 1.61) | (0.45 - 1.64) | (0.60 - 4.48) | (1.05 - 5.30) | (0.17 - 2.69) | (0.51 - 2.23) | (0.09 - 1.84) | (0.43 - 1.84) | (0.32 - 2.25) | (0.29 - 0.89) | (0.52 - 2.25) |
| Hard physical labour: - yes, strongly (Ref.: no, not at all) | 0.97 | 0.66 | 0.63 | 0.62 | 0.47 | 0.75 | 1.29 | 0.77 | 0.42+ | 1.76 | 1.03 | 0.64 |
|  | (0.52 - 1.80) | (0.31 - 1.42) | (0.29 - 1.41) | (0.24 - 1.59) | (0.15 - 1.46) | (0.24 - 2.37) | (0.49 - 3.38) | (0.21 - 2.79) | (0.15 - 1.14) | (0.69 - 4.51) | (0.51 - 2.05) | (0.29 - 1.42) |
| - yes, somewhat | 0.83 | 1.05 | 0.72 | 0.68 | 0.90 | 0.54 | 0.96 | 0.79 | 0.84 | 1.74+ | 1.38 | 0.74 |
|  | (0.50 - 1.36) | (0.64 - 1.72) | (0.38 - 1.36) | (0.34 - 1.35) | (0.37 - 2.16) | (0.22 - 1.32) | (0.44 - 2.07) | (0.33 - 1.88) | (0.42 - 1.70) | (0.92 - 3.27) | (0.82 - 2.32) | (0.45 - 1.23) |
| Workplace bullying: - Often (Ref.: never) | 0.90 | 0.91 | 0.66 | 2.73+ | 1.00 | 0.37 | 0.32 | 0.41 | 2.12 | 0.76 | 1.18 | 0.68 |
|  | (0.31 - 2.65) | (0.36 - 2.34) | (0.23 - 1.91) | (0.92 - 8.12) | (0.25 - 3.94) | (0.11 - 1.27) | (0.07 - 1.47) | (0.11 - 1.45) | (0.49 - 9.10) | (0.24 - 2.45) | (0.35 - 4.04) | (0.25 - 1.83) |
| - Sometimes | 0.91 | 0.97 | 0.47 | 1.57 | 0.80 | 0.55 | 0.32 | 0.24* | 2.26 | 0.67 | 0.90 | 0.83 |
|  | (0.31 - 2.73) | (0.37 - 2.56) | (0.16 - 1.43) | (0.51 - 4.80) | (0.19 - 3.45) | (0.15 - 2.00) | (0.07 - 1.56) | (0.06 - 0.94) | (0.50 - 10.12) | (0.20 - 2.28) | (0.26 - 3.17) | (0.30 - 2.26) |
| - Seldom | 1.42 | 0.69 | 0.76 | 0.40* | 0.35* | 0.75 | 14.23* | 1.60 | 2.42 | 1.12 | 1.81 | 1.36 |
|  | (0.62 - 3.23) | (0.31 - 1.54) | (0.34 - 1.72) | (0.17 - 0.98) | (0.13 - 0.96) | (0.25 - 2.20) | (1.63 - 124.22) | (0.46 - 5.51) | (0.72 - 8.10) | (0.38 - 3.29) | (0.70 - 4.66) | (0.57 - 3.20) |
| Constant | 3.17 | 4.29+ | 0.09** | 0.03*** | 0.17 | 0.04* | 0.01*** | 0.26 | 0.01*** | 0.12* | 0.06** | 0.19* |
|  | (0.70 - 14.43) | (0.91 - 20.12) | (0.01 - 0.54) | (0.00 - 0.22) | (0.02 - 1.84) | (0.00 - 0.54) | (0.00 - 0.13) | (0.02 - 3.17) | (0.00 - 0.08) | (0.01 - 0.93) | (0.01 - 0.37) | (0.04 - 0.96) |
|  |  |  |  |  |  |  |  |  |  |  |  |  |
| Observations | 645 | 572 | 642 | 577 | 645 | 577 | 642 | 561 | 642 | 577 | 645 | 579 |
| Pseudo R² | 0.083 | 0.068 | 0.160 | 0.253 | 0.109 | 0.104 | 0.093 | 0.064 | 0.131 | 0.087 | 0.059 | 0.052 |

All estimates include age, marital status, educational level, smoking status, BMI category, impairments (climb stairs; everyday tasks) and morbidity as potential confounders. Odds Ratios were reported; 95%-confidence intervals in parentheses; *** p<0.001, ** p<0.01, * p<0.05, + p<0.10
